# Association between handgrip strength and metabolic syndrome: A meta-analysis and systematic review

**DOI:** 10.3389/fnut.2022.996645

**Published:** 2022-12-01

**Authors:** Yu Wen, Tiancong Liu, Changcheng Ma, Jianwei Fang, Zhiying Zhao, Mengrui Luo, Yang Xia, Yuhong Zhao, Chao Ji

**Affiliations:** ^1^Key Laboratory of Precision Medical Research on Major Chronic Disease, Shenyang, Liaoning, China; ^2^Department of Clinical Epidemiology, Shengjing Hospital of China Medical University, Shenyang, China; ^3^Department of Otorhinolaryngology - Head and Neck Surgery, Shengjing Hospital of China Medical University, Shenyang, China; ^4^Department of Clinical Laboratory, Shengjing Hospital of China Medical University, Shenyang, China; ^5^Clinical Research Center, Shengjing Hospital of China Medical University, Shenyang, China

**Keywords:** absolute HGS, dose–response analysis, metabolic syndrome, meta-analysis, relative HGS, systematic review

## Abstract

**Background:**

Although muscle strength has been reported to be associated with metabolic syndrome (MetS), the association is still controversial. Therefore, the purpose of this meta-analysis was to identify the association between handgrip strength (HGS) and MetS.

**Methods:**

Original research studies involving HGS and MetS from database inception to 20 May 2022 were selected from PubMed, Web of Science, Embase, China National Knowledge Infrastructure, Wanfang databases, and Chinese Biomedical Document Service System. The odds ratios (ORs) with 95% confidence intervals (CIs) of MetS for HGS were calculated using a random-effects model. A dose–response analysis was performed. Subgroup analysis and meta-regression were also conducted.

**Results:**

Thirty effect sizes (reported in 19 articles) with a total of 43,396 participants were included in this meta-analysis. All studies were considered to be of moderate-to-good quality. An inverse association between HGS (low vs. high) with MetS was shown (OR: 2.59, 95% CI: 2.06−3.25). Subgroup analyses demonstrated the pooled ORs of relative HGS (HGS/weight), relative HGS (HGS/BMI), and absolute HGS were 2.97 (95% CI: 2.37−3.71), 2.47 (95% CI: 1.08−5.63), and 1.34 (95% CI: 1.06−1.68), respectively. Dose–response analysis revealed a significant linear dose–response relationship between relative HGS (HGS/weight) and MetS in observational studies (0.1 HGS/weight: OR, 0.68; 95% CI: 0.62−0.75). Univariate meta-regression analysis indicated that country status, measuring tools of HGS, components of MetS, and diagnosed criteria of MetS explained 16.7%, 26.2%, 30.1%, and 42.3% of the tau-squared in the meta-regression, respectively.

**Conclusion:**

The results of the current meta-analysis indicated that lower HGS is associated with a higher risk of MetS. A linear dose–response association between lower relative HGS (HGS/weight) and increased prevalence of MetS was found. Accordingly, a lower HGS is a significant predictor of MetS.

**Systematic review registration:**

[https://www.crd.york.ac.uk/PROSPERO/], identifier [CRD42021276730].

## Introduction

There are a number of definitions of metabolic syndrome (MetS) according to different organizations. National Cholesterol Education Program Adult Treatment Panel III (NCEP ATP III) defines MetS as having three or more of the following five metabolic risk factors: elevated waist circumference (WC), triglycerides (TG), reduced high-density lipoprotein cholesterol (HDL-C), elevated blood pressure (BP), and elevated fasting blood glucose (FBG) (1). American Heart Association (AHA)/the National Heart, Lung, and Blood Institute (NHLBI) statement maintains the NCEP ATP III criteria except for minor modifications in 2005 (2). According to the International Diabetes Federation (IDF) criteria (3), people with MetS are defined by having central obesity along with two or more of the following abnormalities: (1) elevated TG; (2) reduced HDL cholesterol; (3) elevated BP; (4) elevated fasting plasma glucose (FPG). MetS has become a major health challenge worldwide with the prevalence increasing in concert with obesity and sedentary lifestyles, thus, influencing 10%−40% of the total population (4). MetS is linked to type 2 diabetes, cardiovascular disease, kidney disease, cancer, and mortality (5–7). Considering the rising prevalence of MetS and healthy outcomes, MetS is a major health issue.

High muscle strength is a protective factor for cardiovascular and metabolic health, and decreased muscle strength may increase the risk of chronic diseases in the elderly (8, 9). Muscle strength can be easily measured by handgrip strength (HGS). HGS, a simple, convenient, and economical indicator, can reflect skeletal muscle strength effectively and reliably (10). In addition, HGS can represent skeletal muscle strength in community-dwelling older people (11). Recent epidemiologic studies have been conducted to determine the association between HGS and MetS. However, the results were inconsistent between different kinds of HGS (absolute and relative HGS) and MetS (12, 13). Several studies (12, 14, 15) indicated that there is an inverse association between relative HGS [HGS/body weight or HGS/body mass index (BMI)] and MetS. However, other studies (16, 17) have reported no significant association between absolute HGS and MetS.

The potential mechanisms were as follows: Skeletal muscles, major sites of glucose uptake and deposition (18), can secrete multiple myokines (such as interleukin-15, myostatin, and irisin) (19). Of these myokines, interleukin-15 is involved in the regulation of fat mass (20). Myostatin can inhibit body fat accumulation and improve insulin sensitivity (21–23). A release of irisin from skeletal muscle negatively regulates glucose homeostasis and obesity (24). Insulin resistance (IR) is a reduced biological response of peripheral tissues to insulin (25, 26). IR plays a major role in MetS (27). Obesity was significantly associated with the increased risk of MetS. IR and obesity are important factors of MetS, muscle strength is related to MetS.

For the inconsistent results and the potential mechanisms, we conducted the first meta-analysis of HGS and MetS for the following purposes: (1) to assess the association between HGS and MetS; (2) to evaluate a dose–response pattern of HGS and MetS; and (3) to provide reliable evidence-based medicine for the prevention and treatment of MetS in the population.

## Methods

This meta-analysis was reported according to the Preferred Reporting Items for Systematic Review and Meta-Analyses statement (28).

This study was registered at https://www.crd.york.ac.uk/PROSPERO/ as CRD42021276730.

### Eligibility criteria

The inclusion criteria were as follows: (1) The definition of MetS is a cluster of metabolic abnormalities, including abdominal obesity, hypertension, elevated FPG, elevated TG, and low HDL-C. MetS was diagnosed according to NCEP ATP III criteria, IDF classification, and AHA criteria or defined by other criteria. (2) The study was published in English or Chinese. (3) The authors analyzed the association between HGS and MetS. (4) an observational study. (5) Evaluation of grip strength was measured by a handheld dynamometer. (6) Participants were ≥18 years of age.

The exclusion criteria were as follows: (1) reports with only abstracts or unpublished results; (2) the types of papers were reviews, secondary analyses, letters, and conferences; (3) duplicated papers from different search sources; (4) duplicated study population (If more than one publication was published on the same population, study with the largest population was selected); (5) the author did not investigate the association between HGS and MetS; (6) unavailable full text; (7) unavailable data [odds ratios (ORs), hazard ratios (HRs) or prevalence rate ratios (PRRs) were not mentioned in the text]; (8) studies reported the separate MetS components (e.g., elevated WC, elevated TG, low HDL-C, elevated BP, and elevated FBG), instead of MetS; and (9) did not report HGS at baseline.

### Search strategy

Original research studies from database inception to 20 May 2022 were selected from PubMed, Web of Science, Embase, Chinese National Knowledge Information (CNKI), Wanfang databases, and Chinese Biomedical Document Service System (SinoMed). A PubMed search for studies involving HGS and MetS was conducted without restrictions by combining synonymous or related search terms for HGS and MetS. The keywords used in the PubMed search were converted to search tags for Web of Science and Embase. The detailed search strategy is shown in Supplementary Table 1.

### Literature screening and data collection

Two independent researchers (YW and ML) working in pairs screened the titles and abstracts of the initially identified studies to determine if the studies satisfied the selection criteria. Any disagreements were discussed and agreed upon through consensus. The retrieved studies were re-assessed by two independent investigators to ensure that the studies satisfied the inclusion criteria.

Two independent researchers (YW and ML) performed data extraction, relevant data were extracted from each included study using a standardized extraction form. The characteristics included author, year, country, study design, follow-up (years), sample size, age, gender, definition of MetS, HGS measuring tools, HGS measuring position, adjusted variables, and quality score. After extraction, we reviewed the data and compared it interactively. If inconsistent data were detected, we would solve discrepancies by researching and discussing the article. The extracted data results are shown in Table 1.

**TABLE 1:** Characteristics of observational studies evaluating the association between handgrip strength (HGS) and metabolic syndrome (MetS) in the meta-analysis.

Authors, year	Country	Study design	Follow-up (years)	Sample size	Age	Gender	Definition of MetS	HGS measuring tools	Effect size (95%CI)	Rates (%)	HGS measuring position	HGS (kg)	Adjusted variables	Quality score
Sayer et al. (46)	United Kingdom	Cross-sectional study	-	2,677	59–73	Men: 1,438 women: 1,239	IDF and NCEP ATP III	A Jamar handgrip dynamometer	OR: 1.18 (1.07, 1.30)	Prevalence: 32.2	Unreported	Men: 44.3 ± 7.4 women: 26.7 ± 5.7	Gender, weight, age, walking speed, social class, smoking habit, and alcohol intake	9
Atlantis et al. (37)	Australia	Cross-sectional study	-	1,195	35−81	Men: 1,195	IDF and NCEP ATP III	A grip dynamometer (Smedley, Chicago, IL)	OR: 2.15 (1.14, 4.04)	Prevalence: 37.7	Unreported	MetS: 13.4 ± 2.0 (kg F/[kg LM arm])Not MetS: 14.3 ± 2.0 (kg F/[kg LM arm])	Unreported	7
Ishii et al. (40)	Japan	Cross-sectional study	-	1,971	≥65	Men: 977 women: 994	NCEP ATP III	A digital grip strength dynamometer (Takei Scientific Instruments, Niigata, Japan)	OR: men: 2.63 (1.32, 5.26) women: 1.85 (1.11, 3.03)	Prevalence: men: 43.6 women: 28.9	Unreported	34.8 ± 6.0	Age, height, weight, physical activity, and food intake	7
Chang et al. (38)	China	Cross-sectional study	-	628	≥65	Men: 305 women: 323	NCEP ATP III	An analog isometric dynamometer (Baseline hydraulic hand dynamometer, Fabrication Enterprises, Inc., Irvington, NY, United States)	OR: 0.24 (0.02, 3.14)	Prevalence: 28.0	Unreported	26.9 ± 15.3	Unreported	6
Kawamoto et al. (41)	Japan	Cross-sectional study	-	1,679	≥50	Men: 742 women: 937	NCEP ATP III	Dynamometer type not reported	OR: men: 6.25 (3.48, 11.22) women: 6.25 (3.95, 9.88)	Prevalence: men: 27.4 women: 47.8	Unreported	26.7 ± 8.4	Age, exercise habits, smoking status, drinking status, low-density lipoprotein cholesterol, eGFR, and medication (such as antihypertensive, antidyslipidemic, and/or antidiabetic medications)	4
Byeon et al. (16)	South Korea	Cross-sectional study	-	1,009	50.3 ± 13.8	Men: 488 women: 521	NCEP ATP III	A handgrip dynamometer (TKK 5401, Japan)	OR: men: 2.52 (1.43, 4.46) women: 5.01 (1.66, 15.08)	Prevalence: men: 22.3 women: 7.5	A standing position	30.5 ± 9.5	Age and weight	6
Wu et al. (14)	China	Cross-sectional study	-	17,703	45.2 (51.3–59.2)	Men: 10,093 women: 7,610	The American Heart Association scientific statements of 2009	A handgrip dynamometer (EH101; CAMRY, Guangdong, China)	OR: men: 3.36 (2.97, 3.80) women: 3.89 (3.22, 4.71)	Prevalence: men: 41.6 women: 22.9	A standing position	MetS: 33.9 (33.8, 34.1)Not MetS: 34.3 (34.2, 34.4)	Age, smoking status, drinking status, physical activity, educational levels, household income, total energy intake, and family history of diseases (including CVD, hypertension, hyperlipidemia, and diabetes)	8
Chang et al. (39)	China	Cross-sectional study	-	129	>65	Men: 60 women: 69	Modified NCEP ATP III for Asians	Dynamometer type not reported	OR: 0.99 (0.88, 1.11)	Prevalence: 20.2	A seated position	MetS: 28.0 ± 8.4Not MetS: 32.0 ± 8.5	Sociodemographic variables, lifestyle factors, total energy intake, and family history of disease	6
Mesinovic et al. (44)	Australia	Cross-sectional study	-	84	≥50	Men: 38 women: 46	IDF	A Jamar Plus Digital hydraulic hand grip dynamometer (Patterson Medical, Bolingbrook, IL, United States)	OR: 0.74 (0.02, 27.11)	Prevalence: 76.2	A seated position	MetS: 30.9 ± 11.0Not MetS: 30.4 ± 9.9	Age and gender	6
Merchant et al. (17)	Singapore	Cross-sectional study	-	722	≥65	Men: 325 women: 397	Modified NCEP ATP III for Asians	A digital dynamometer (A5401, Takei Scientific Instruments, Co., Ltd., Japan)	OR: 1.96 (1.64, 2.33)	Prevalence: 41.0	a seated position	22.5 ± 6.9	Age, years of education, duration of exercise, history of smoking, and alcohol consumption	8
Ji et al. (15)	America	Cross-sectional study	-	5,056	≥20	Men: 2,535 women: 2,521	The joint scientific statement of harmonizing the MetS criteria	A Takei Digital Grip Strength Dynamometer (T.K.K. 5401)	OR: men: 7.69 (4.87, 12.16) women: 8.33 (5.56, 12.50)	Prevalence: men: 36.4 women: 36.3	A standing position	Unreported	Age, race, drinking status, smoking status, education level, income, total energy intake, and physical activity	7
Song et al. (47)	China	Cross-sectional study	-	909	≥60	Men: 385 women: 524	IDF	A handheld dynamometer (DRIP-D; Takei, Ltd., Niigata, Japan)	OR: men: 3.17 (1.78, 5.65) women: 1.78 (1.22, 2.60)	Prevalence: men: 26.8 women: 46.9	A standing position	Men: MetS: 32.3 ± 7.4Not MetS: 31.6 ± 7.4Women: MetS: 20.1 ± 5.4Not MetS: 19.7 ± 5.5	Age, smoking status, drinking status, occupation, educational level, family income, nutritional status, and physical activity level	9
Shen et al. (51)	China	Cohort study	4 years	3,350	≥45	Men: 1,845 women: 1,505	Modified NCEP ATP III for Asians	A hand-held dynamometer (WCS-100, Nantong, China)	HR: men: 1.76 (1.12, 2.78) women: 1.28 (1.03, 1.55)	Incidence: men: 11.8 women: 24.1	A standing position	Men: MetS: 39.1 ± 8.9Not MetS: 37.4 ± 8.8Women: MetS: 26.4 ± 6.5Not MetS: 26.4 ± 6.6	Age, marital status, region, education, current smoker, alcohol consumption, hypertension, hyperlipidemia, diabetes, and waist circumstance	9
Moreira et al. (45)	Brazil	Cross-sectional study	-	419	40−65	Women: 419	NCEP ATP III	A Jamar handgrip dynamometer	PRR: 1.31 (1.15, 1.50)	Prevalence: 65.6	A seated position	0.92 ± 0.22 Kgf/(kg/m^2^)	Unreported	9
Kim et al. (42)	South Korea	Cross-sectional study	-	256	40−69	Men: 256	NCEP ATP III	A Takei 5401 grip dynamometer (Takei, Co., Ltd., Japan)	OR: 4.76 (2.09, 8.81)	Prevalence: 30.5	A standing position	MetS: 36.2 ± 6.4Not MetS: 37.7 ± 6.5	Age, alcohol consumption, and smoking.	6
Shen (51)	China	Cross-sectional study	-	3,598	≥60	Men: 1,267 women: 2,331	Chinese Diabetes Society	A handheld dynamometer (WCS-100)	OR: men: 3.72 (2.60, 5.30) women: 3.00 (2.36, 3.82)	Prevalence: men: 38.6 women: 52.8	Unreported	Men: MetS: 31.2 ± 8.1Not MetS: 30.7 ± 7.7Women: MetS: 18.0 ± 5.6Not MetS: 18.3 ± 5.4	Age, race, educational level, smoking, and drinking	7
Kim and Seo (43)	South Korea	Cross-sectional study	-	1,096	≥45	Men: 435 women: 661	The American Heart Association criteria (2005)	A digital grip strength dynamometer (Takei TKK5401; Takei Scientific Instruments, Niigata, Japan)	OR: men: 3.87 (1.73, 8.65) women: 3.53 (1.94, 6.40)	Prevalence: men: 39.9 women: 46.4	A standing position	54.2 ± 0.7	Age, physical activity, high-risk drinking, and cancer type	7
Jeon et al. (50)	South Korea	Cohort study	16 years	2,538	40−69	Men: 1,323 women: 1,215	NCEP ATP III	A digital grip dynamometer (Grip-D T.K.K.5401 and T.K.K.5102, TAKEI Science Instruments, Co., Ltd., Nigata, Japan)	HR: men: 2.51 (1.94, 3.26) women: 2.70 (1.96, 3.72)	Incidence: men: 41.4 women: 36.4	Unreported	Men: MetS: 35.0 ± 5.8Not MetS: 35.4 ± 5.5Women: MetS: 21.4 ± 3.8Not MetS: 22.0 ± 3.8	Muscle strength plus age; family history of DM (yes, no); family history of hypertension (yes, no); job (homemaker, white collar, or blue collar); income (KRW < 1,000,000, KRW 1,000,000 ≤ or < 2,000,000, KRW 2,000,000 ≤ or < 4,000,000, or KRW 4,000,000 ≤); education (<12 years and ≥12 years); married status (yes, no); smoker (never smoker, ex-smoker, or current smoker); alcohol consumption (g/day); and leisure physical activity (METs). Women added menopause (yes and no)	7
Zhang et al. (48)	China	Cross-sectional study	–	1,054	≥40	Women: 1,054	NCEP ATP III	A grip dynamometer (TKK5401, Takei, Japan)	OR: middle-aged: 1.55 (1.18, 4.31) elderly: 1.24 (1.05, 2.12)	Prevalence: middle-aged: 21.2 elderly: 39.4	A standing position	Middle-aged: MetS: 24.5 ± 4.5Not MetS: 22.9 ± 4.1Elderly: MetS: 22.9 ± 3.6Not MetS: 21.0 ± 3.9	Age, alcohol consumption, smoking status, and physical activity	7

ATP III, National Cholesterol Education Program Adult Treatment Panel III; HGS, handgrip strength; IDF, International Diabetes Federation; MetS, metabolic syndrome.

### Quality assessment

Two researchers (YW and ML) independently assessed the quality of each study by the quality assessment tools. An 11-item checklist of the Agency for Healthcare Research and Quality (AHRQ) (29) was used to assess the quality of cross-sectional studies. An item was scored “0” if the item was answered “NO” or “UNCLEAR;” if the item was answered “YES,” then the item scored “1.” Article quality was assessed as follows: low quality = 0−3; moderate quality = 4−7; and high quality = 8−11. The Newcastle-Ottawa Scale (NOS) (30) was used to evaluate the quality of a cohort or case-control study. A total of 9 points from 3 domains were reported for each study [selection, comparability, and ascertainment of exposure(s) or outcome(s)]. No case-control study was identified for this review. Thus, the NOS was performed for cohort studies only.

### Statistical analysis

In this meta-analysis, we considered that HRs were approximately equivalent to ORs when the prevalence of the outcome was low (31, 32). In addition, the prevalence rate ratio (PRR) was also assumed approximately equivalent to OR (33). The exponential transformation was used to convert β to OR. Therefore, we combined OR, HR, and PRR. *Q*-statistics and *I*^2^ statistics were calculated to evaluate heterogeneity in these studies. A *P*-value < 0.05 for *Q*-statistics and *I*^2^ > 50% were regarded to be significant heterogeneity. A random effect model was used to pool ORs for participants due to considerable heterogeneities among studies. Sensitivity analysis was performed to assess the influence of a single study on the overall combined results. Egger’s test was used to evaluate publication bias. To address publication bias, trim-and-fill adjusted analysis (34) was applied. We explored potential heterogeneity in estimates of the treatment effects using univariate meta-regression (35).

This study performed subgroup analyses by gender, country status (developing or developed country according to International Monetary Fund^1^), study type, age, publication year, the diagnosed criteria of MetS (NCEP ATP III classification, IDF classification, and AHA criteria), HGS-adjusted methods (absolute HGS; HGS/body weight; HGS/BMI), measuring tools of HGS, and components of MetS.

We used the method described by Greenland and Longnecker (36) to conduct a dose–response analysis. The assigned dose was the midpoint of the upper and lower boundaries of each HGS category. The distributions of cases and non-cases for at least three categories of exposure were also collected. A dose–response meta-analysis was performed. And the *glst* order was applied to carry out the model estimation and draw a linear dose–response association plot. All of the statistical analyses were performed using Stata software (version 11.2; Stata Corp., College Station, TX, United States).

## Results

### Study selection

**FIGURE 1:**
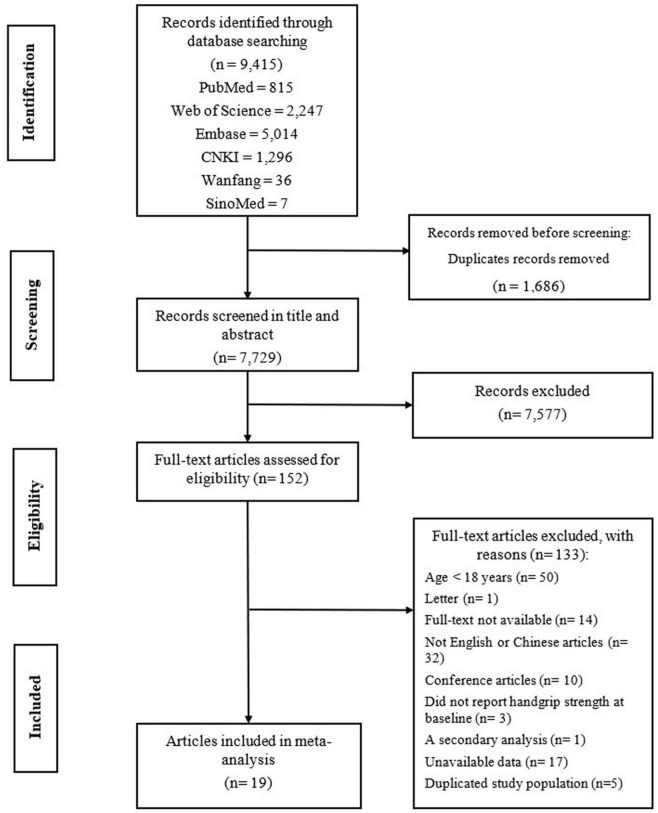
Flow chart of the literature search and selection process.

Figure 1 indicates the literature search process. Our initial search identified 9,415 papers, 1,686 of which were duplicates, thus, yielding 7,729 articles. After screening, 7,577 articles were excluded because the articles did not meet the inclusion criteria. Of the 152 articles assessed for eligibility, 133 were excluded for the reason that the age of participants in 50 articles was <18 years of age, 1 article was a letter, full texts of 14 articles were unavailable, 32 articles were not in English or Chinese, 10 articles were conferences, 3 articles did not report HGS at baseline, 1 article was a secondary analysis, 17 articles were unavailable data, and 5 articles have duplicated study population. Finally, our meta-analysis included 19 articles.

### Study characteristics

Table 1 shows the study characteristics. Of the 30 studies in 19 articles, 26 (12, 14, 15, 17, 37–50) were cross-sectional studies and 4 (50, 51) were cohort studies. A total of 24 studies in 14 articles (12, 14, 17, 38–43, 47–51) originated from Asia [7 studies in 4 articles (14, 42, 43, 50) from South Korea, 12 studies in 7 articles (12, 38, 39, 47–49, 51) from China, 4 studies in 2 articles (40, 41) from Japan, and 1 study (17) from Singapore], 2 studies in an article (15) from the United States, 2 studies (37, 44) from Australia, 1 study (46) from United Kingdom, and 1 study (45) from Brazil. The total sample size was 43,396 in the 30 studies (reported in 19 articles). The age of the study population was = 18 years.

Jamar [3 studies (44–46)], Takei [16 studies in 9 articles (14, 15, 17, 40, 42, 43, 47, 48, 50)], Smedley [1 study (37)], Camry [1 study (12)], WCS-100 [4 studies in 2 articles (49, 51)], and hydraulic hand dynamometer [1 study (38)] were used to measure HGS. There were 4 studies in 2 articles (39, 41) that did not specify the name of the handgrip dynamometer. HGS measurement positions included standing [13 studies in 8 articles (12, 14, 15, 42, 43, 47, 48, 51)] and seated positions [4 studies (17, 39, 44, 45)]. A total of 13 studies in 7 studies (37, 38, 40, 41, 46, 49, 50) did not introduce the measurement positions of HGS.

National Cholesterol Education Program Adult Treatment Panel III was the most commonly used criterion to evaluate HGS in the analyzed publications. As shown in Table 1, MetS was defined by the NCEP ATP III classification (*n* = 13) (14, 17, 37–42, 45, 46, 48, 50, 51), IDF classification (*n* = 2) (44, 47), AHA classification (*n* = 2) (12, 43), or other criteria (*n* = 2) (15, 49).

### Quality assessment of included studies

Results of the quality assessment of the included studies are shown in Table 1 and Supplementary Tables 2, 3. We used the total score of this assessment to evaluate articles of insufficient quality. No study was excluded based on the AHRQ and NOS, as all of them were considered to be of moderate-to-good quality.

### Handgrip strength and metabolic syndrome

Several studies analyzed the association between absolute HGS and MetS. In these studies, 4 studies demonstrated that absolute HGS was not significantly associated with MetS (38, 44, 48, 50). Another 4 studies found that lower absolute HGS had an increased likelihood of having MetS (37, 39, 40, 46). In one Chinese study, Qiu-Feng et al. (52) found that a positive association existed between absolute HGS and MetS in male residents, while the association was opposite in female residents.

A total of 12 studies investigated the association between relative HGS (HGS/weight) and MetS. 11 studies demonstrated that relative HGS (HGS/weight) was inversely associated with MetS (12, 15, 17, 41, 42, 47–52). In a total of 3,598 Chinese community residents over 60 years old, a significant negative correlation between HGS/weight and the risk of MetS, regardless of sex (49). However, no significant association was observed in the 628 community-dwelling elders (38).

5 studies assessed the association between relative HGS (HGS/BMI) and MetS. The results showed HGS/BMI were negatively associated with MetS (17, 43, 45, 47, 50).

**FIGURE 2:**
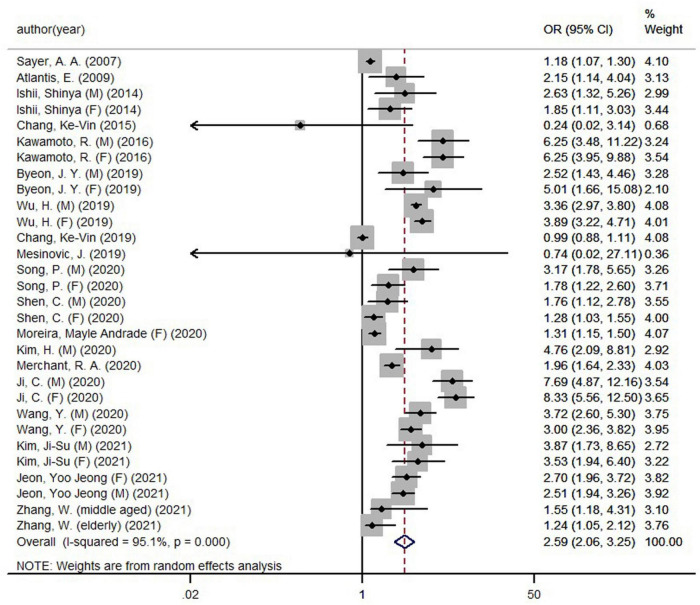
Forest plot of handgrip strength and metabolic syndrome.

Figure 2 demonstrated the pooled OR (low vs. high) of the association between HGS (absolute HGS, HGS/body weight, and HGS/BMI) and MetS was 2.59 (95% CI, 2.06−3.25) in the meta-analysis, with evidence of significant heterogeneity (*I*^2^ = 95.1%, *P* < 0.05). The results illustrated an inverse association of HGS with MetS in adults.

### Subgroup analysis

In this meta-analysis, we performed a subgroup analysis according to gender, age, publication year, HGS-adjusted methods, country status, study type, MetS for each component, and defined criteria of MetS. Compared with participants with high HGS, the ORs (95% CIs) for MetS in participants with low HGS were 3.33 (2.69, 4.14) and 2.57 (1.82, 3.64) in men and women, respectively (Table 2). Compared with participants with high HGS, the ORs (95% CIs) for MetS in participants with low HGS were 2.03 (1.65−2.51), 3.51 (3.18−3.89), 2.20 (1.37−3.53), 8.04 (5.94, 10.89), and 3.21 (2.63, 3.92) according to the NCEP ATP III criteria, AHA criteria, IDF criteria, the joint scientific statement of harmonizing the MetS criteria, and Chinese Diabetes Society, respectively (Table 2). In addition, subgroup analysis by the HGS-adjusted methods suggested that the risks of MetS among adults were negatively associated with absolute HGS (OR, 1.34; 95% CI, 1.06−1.68), relative HGS (HGS/weight; OR, 2.97; 95% CI, 2.37−3.71), and relative HGS (HGS/BMI; OR, 2.47; 95% CI, 1.08−5.63; Table 2). In developed countries, HGS (low vs. high) was significantly and negatively associated with the risk of MetS (OR, 3.29; 95% CI, 2.32−4.68). Similarly, in developing countries, HGS (low vs. high) was significantly and negatively associated with the risk of MetS (OR, 1.75; 95% CI, 1.20−2.56; Table 2). Subgroup analysis based on study type showed a negative association between HGS (low vs. high) and MetS in cross-sectional studies (OR, 2.72; 95% CI, 2.10−3.52) and cohort studies (OR, 1.97; 95% CI, 1.31−2.95; Table 2). A subgroup analysis based on age indicated that there was an inverse association between HGS (low vs. high) and MetS in the middle-aged (OR, 1.32; 95% CI, 1.16−1.50) and elderly population (OR, 1.92; 95% CI, 1.26−2.92). According to the measuring tools of HGS, we divided all studies into four subgroups. The results of four subgroups, HGS (low vs. high) measured by Jamar (OR, 1.22; 95% CI, 1.13−1.32), Takei (OR, 2.88; 95% CI, 2.20−3.77), CAMRY (OR, 3.55; 95% CI, 3.09−4.08), and WCS-100 (OR, 2.23; 95% CI, 1.30−3.83), demonstrated that HGS (low vs. high) was inversely associated with MetS. Among the subgroup analysis of publication year, there was a negative association between HGS (low vs. high) and MetS in 5 years ago (OR, 2.44; 95% CI, 1.24−4.81) and in recent 5 years (OR, 2.60; 95% CI, 2.01−3.37). Among a subgroup based on the components of MetS, an inverse correlation was shown in the association of HGS (low vs. high) with elevated WC (OR, 4.35; 95% CI, 2.74−6.92), elevated TG (OR, 1.69; 95% CI, 1.38−2.05), HDL-C (OR, 1.55; 95% CI, 1.27−1.88), elevated BP (OR, 1.55; 95% CI, 1.30−1.85), and elevated FBG (OR, 1.55; 95% CI, 1.28−1.88).

**TABLE 2:** Combined results of subgroup analysis of HGS and MetS.

Subgroup analysis	Pooled OR (95%CI), *P*-value for the heterogeneity *Q* test, *I*^2^ statistics (%), number of estimates in included studies (n)	
	*n*	Risk estimates of MetS
Sex		
Men	12	3.33 (2.69, 4.14); *I*^2^ = 67.7%, *P* = 0.000
Women	13	2.57 (1.82, 3.64); *I*^2^ = 94.1%, *P* = 0.000
Diagnosed criteria of MetS		
ATP III	19	2.03 (1.65, 2.51); *I*^2^ = 90.7%, *P* = 0.000
AHA	4	3.51 (3.18, 3.89); *I*^2^ = 0.0%, *P* = 0.646
IDF	3	2.20 (1.37, 3.53); *I*^2^ = 33.5%, *P* = 0.222
The joint scientific statement of harmonizing the MetS criteria	2	8.04 (5.94, 10.89); *I*^2^ = 0.0%, *P* = 0.798
Chinese Diabetes Society	2	3.21 (2.63, 3.92); *I*^2^ = 0.0%, *P* = 0.327
HGS adjusted method		
Absolute HGS	6	1.34 (1.06, 1.68); *I*^2^ = 74.0%, *P* = 0.002
HGS adjusted by weight	21	2.97 (2.37, 3.71); *I*^2^ = 90.1%, *P* = 0.000
HGS adjusted by BMI	3	2.47 (1.08, 5.63); *I*^2^ = 87.7%, *P* = 0.000
Country Status		
Developed country	17	3.29 (2.32, 4.68); *I*^2^ = 93.5%, *P* = 0.000
Developing country	13	1.95 (1.39, 2.75); *I*^2^ = 96.5%, *P* = 0.000
Study design		
Cross-sectional study	26	2.72 (2.10, 3.52); *I*^2^ = 95.6%, *P* = 0.000
Cohort study	4	1.97 (1.31, 2.95); *I*^2^ = 87.0%, *P* = 0.000
Age		
Middle-aged	2	1.32 (1.16, 1.50); *I*^2^ = 0.0%, *P* = 0.618
Elderly	7	1.92 (1.26, 2.92); *I*^2^ = 94.4%, *P* = 0.000
Measuring tools of HGS		
Jamar	3	1.22 (1.13, 1.32); *I*^2^ = 0.0%, *P* = 0.445
Takei	16	2.88 (2.20, 3.77); *I*^2^ = 84.1%, *P* = 0.000
CAMRY	2	3.55 (3.09, 4.08); *I*^2^ = 37.7%, *P* = 0.205
WCS-100	4	2.23 (1.30, 3.83); *I*^2^ = 92.7%, *P* = 0.000
Publication year		
before five years	7	2.44 (1.24, 4.81); *I*^2^ = 93.0%, *P* = 0.000
recent five years	23	2.60 (2.01, 3.37); *I*^2^ = 95.1%, *P* = 0.000
Component of MetS		
Elevated waist circumference	11	4.35 (2.74, 6.92); *I*^2^ = 95.5%, *P* = 0.000
Elevated triglycerides	9	1.69 (1.38, 2.05); *I*^2^ = 76.9%, *P* = 0.000
Low high-density lipoprotein-cholesterol	10	1.55 (1.27, 1.88); *I*^2^ = 74.6%, *P* = 0.000
Elevated blood pressure	9	1.55 (1.30, 1.85); *I*^2^ = 75.3%, *P* = 0.000
Elevated fasting blood glucose	9	1.55 (1.28, 1.88); *I*^2^ = 75.2%, *P* = 0.000

ATP III, National Cholesterol Education Program Adult Treatment Panel III; HGS, handgrip strength; IDF, International Diabetes Federation; AHA, the American Heart Association criteria; MetS, metabolic syndrome; BMI, body mass index.

### Dose–response analysis

**FIGURE 3:**
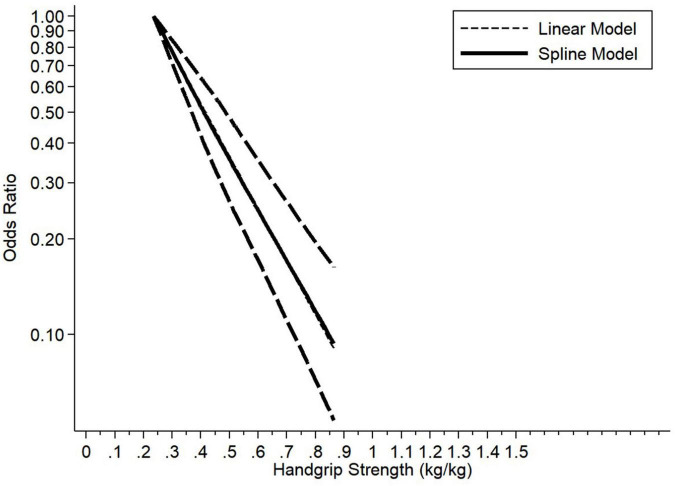
Dose–response association between handgrip strength (HGS/weight) and prevalence of metabolic syndrome.

Three articles were included for the dose–response meta-analysis of the association between relative HGS (HGS/weight) and risk of MetS. There was a negative linear association between relative HGS (HGS/weight) and MetS (0.1 HGS/weight: OR, 0.68; 95% CI, 0.62−0.75) in Figure 3.

### Publication bias and sensitivity analysis

Egger’s test indicated the existence of publication bias (*P* < 0.05) among the total group of subjects. Therefore, Duval and Tweedie’s trim-and-fill method was performed to re-examine publication bias. The adjusted pooled OR after adding nine studies was OR was 1.76 (95% CI: 1.40−2.21, *P* < 0.05). Hence, publication bias was solved when nine studies were incorporated in the funnel plot by trim fill analysis (Supplementary Figure 1). After excluding each article in every turn, the combined results were stable in the sensitivity analysis and the pooled ORs (95% CIs) ranged between 2.47 (1.98, 3.09) and 2.69 (2.15, 3.37).

### Meta-regression

Examination of the association of HGS and MetS in adults revealed high study heterogeneity (*I*^2^ = 95.1%, *P* < 0.05). Thus, we conducted a meta-regression to identify the sources of high heterogeneity. Univariate meta-regression analysis indicated that country status (*P* = 0.025), measuring tools of HGS [Takei (*P* = 0.024) and CAMRY (*P* = 0.029)], components of MetS [elevated WC (*P* = 0.000)], and diagnosed criteria of MetS [ATP III (*P* = 0.001) and IDF (*P* = 0.016)] were significant sources of heterogeneity. Country status, measuring tools of HGS, components of MetS (elevated TG as the reference), and diagnosed criteria of MetS explained 16.7%, 26.2%, 30.1%, and 42.3% of the tau-squared in the meta-regression, respectively. Sex, HGS-adjusted method, study design, age, and publication year were not the potential sources of heterogeneity. The results are shown in Supplementary Table 4.

## Discussion

This meta-analysis was the first study to systematically evaluate the association between HGS and MetS. In this meta-analysis involving 30 studies (reported in 19 articles) with a total of 43,396 participants, we determined the association between HGS and MetS. The study types of included studies were cross-sectional studies and cohort studies. Additionally, we used AHRQ and NOS scales to assess the quality of our included studies, seven studies were rated high quality and twelve studies were rated moderate quality. Most cross-sectional studies did not mention relevant contents of follow-up. As a whole, all studies reached the criteria of quality assessment. Based on cross-sectional and prospective cohort studies, this meta-analysis of observational studies indicated there was an inverse association between HGS and MetS. Low HGS was associated with a 2.59-fold increase in the pooled prevalent MetS. Consistent results were demonstrated based on subgroup analysis regarding gender, age, publication year, HGS-adjusted methods, region country status, study type, MetS for each component, and defined criteria of MetS. Due to significant heterogeneity among the included studies, we performed meta-regression and subgroup analysis to investigate the potential sources of heterogeneity. Meta-regression analysis revealed that country status, measuring tools of HGS, components of MetS, and diagnosed criteria of MetS contributed to the heterogeneity. However, the heterogeneity was still uncontrolled after subgroup analyses. Furthermore, in the dose–response analysis, a linear association between relative HGS (HGS/body weight) levels and MetS was found among men and women. These results demonstrated that any incremental increase in the relative HGS level was linked to a decreased prevalence in the MetS.

The pathogenesis of MetS is partly understood; however, a sedentary lifestyle, unhealthy diet, overweight or obesity, and still largely unknown genetic factors clearly interact to produce it (53). Regular physical activity has been shown to decrease body weight and visceral fat (54, 55) accumulation and improves insulin sensitivity (56, 57). Those are major constituents or related to MetS. A cohort study of 612 middle-aged men shows that high-intensity leisure-time physical activity at least 3 h/week was half as likely as sedentary to develop the MetS during the 4 years follow-up period (58). However, physical activity seems to be difficult to assess accurately (59). HGS has been introduced as a simple and inexpensive assessment tool for muscle strength and is highly correlated with total muscle strength (60). As a partly function of resistance training, physical activity could increase muscle strength (48). Low physical activity causes a decrease in muscle strength, which is known to increase MetS. And the mechanisms of physical activity and muscle strength were very similar. Both of them could decrease plasma triglyceride concentrations and increase plasma HDL cholesterol concentrations, improving insulin sensitivity (53). HGS is an essential indicator of physical fitness and can reflect total muscle strength (59). Moreover, HGS is much easier to assess. Therefore, HGS is a flexible indicator with which to screen MetS. Several epidemiologic studies (12, 15, 37) showed an adverse association between relative HGS and MetS. A cross-sectional study conducted in Tianjin showed that relative HGS is inversely associated with MetS in a survey of 17,703 individuals > 40 years of age (12). Another study conducted in the United States suggested that increased relative HGS may be inversely related to the prevalence of MetS or its separate components using 2011−2014 National Health and Nutrition Examination Survey (NHANES) data (15). An Australian population-based study indicated that there was a negative association between muscle strength and MetS (37). Previous studies (14, 17) that investigated the association between absolute HGS and MetS were not in agreement with relative HGS (HGS/weight or HGS/BMI). No association between absolute HGS and MetS was detected among 1,009 Korean participants (14). A previous cross-sectional study reported that there was no association between absolute HGS and MetS in older adults from the HOPE study (17). Moreover, no dose–response meta-analysis between relative HGS (HGS/weight) and MetS was conducted. The dose–response meta-analysis results indicated that there was a negative linear association between relative HGS (HGS/weight) and MetS.

In agreement with previous studies, we found that a lower relative HGS (HGS/body weight; HGS/BMI) was associated with a higher prevalence of MetS. Wu et al. (14) revealed that physical inactivity, energy intake, and partial pathophysiologic mechanisms influenced muscle weakness in MetS. First, insulin acts on skeletal muscle and plays a significant role in IR and insulin sensitivity (61–63). Insulin sensitivity and IR are important factors in the pathophysiology of metabolic disorders (61, 62). Second, skeletal muscle secretes muscle factors that have an endocrine role, such as irisin and myonectin (64–67). The above processes are blocked and become a risk factor for MetS (68, 69). Third, in skeletal muscle, excessive accumulation of intramuscular fat will cause IR and develop into MetS (13, 70). Fourth, several studies have shown that lower muscle strength and mass may be associated with higher inflammatory markers (71), thereby increasing the risk of MetS. Therefore, skeletal muscle releases muscle factors and reduces inflammatory markers, decreasing the prevalence of MetS.

The current meta-analysis had several strengths. First, this is the first meta-analysis and systematic review to assess the association between HGS and MetS. Second, we reconciled the inconsistent results of absolute HGS and relative HGS (HGS/weight; HGS/BMI). Third, based on our meta-analysis, despite the limited number of included studies, we evaluated the dose–response association between relative HGS (after adjustment for body weight) and MetS for men and women.

Of note, there were some potential limitations in our meta-analysis. First, the insufficient reporting limited the interpretability of the results. Second, because we solely searched the literature published in English or Chinese, it was likely that all pertinent results were not included. Finally, HRs were treated as equal to ORs for pooling and analyzing data, which may have impacted the results.

## Conclusion

In summary, the results of the current meta-analysis indicate that higher HGS is associated with a lower risk of MetS. Moreover, there appears to be a negative dose–response association between relative HGS (HGS/weight) and MetS. Lower levels of relative HGS (HGS/weight) were associated with a higher prevalence of MetS. Accordingly, lowered relative HGS (HGS/weight) might be a significant predictor of MetS.

## Data availability statement

The original contributions presented in this study are included in the article/Supplementary material, further inquiries can be directed to the corresponding author/s.

## Author contributions

YW did the conceptualization, carried out the software and formal analysis, extracted the data, and wrote the original draft. TL, CM, JF, and ZZ wrote, reviewed, and edited the manuscript. ML wrote, reviewed, and edited the manuscript and extracted the data. YX wrote, reviewed, and edited the manuscript and supervised the data. YZ wrote, reviewed, and edited the manuscript and carried out the funding acquisition. CJ did the conceptualization, wrote, reviewed, and edited the manuscript, supervised the data, and carried out the funding acquisition. All authors contributed to the article and approved the submitted version.

## Footnotes

1
https://www.imf.org/external/pubs/ft/weo/2020/01/weodata/groups.htm

## Supplementary material

Supplementary Figure 1 Adjusted effect size using trim and fill method for HGS and MetS.

Supplementary Table 1 Search strategy.

Supplementary Table 2 The quality assessment of the included cohort studies.

Supplementary Table 3 The quality assessment of the included cross-sectional studies.

Supplementary Table 4 Meta-regression analysis of HGS and MetS.
