# Supplementary material for: Association between handgrip strength and metabolic syndrome: A meta-analysis and systematic review
Source: Front Nutr. 2022 Dec 1;9:996645. doi: 10.3389/fnut.2022.996645 (PMC9751936; doi:10.3389/fnut.2022.996645)
Supplement: Supplementary Figure 1 — Adjusted effect size using trim and fill method for HGS and MetS. [file Data_Sheet_1.ZIP › Supplementary material/Table S1. Search strategy.docx]

**Table S1. Search strategy of HGS and MetS**

| Database | Search | Search Strings | Hits |
| --- | --- | --- | --- |
| PubMed | #1 | "hand strength" OR "handgrip" OR "handgrip strength" OR "grip strength" OR "holding power" OR "hand strengths" OR "strength hand" OR "strengths hand" OR "grip" OR "grips" OR "grasp" OR "grasps" OR "hand grip" OR "grip power" OR "grip-strength" OR "muscle strength" OR "muscle strength Dynamometer" OR "grip dynamometer" OR "muscle weakness" OR "muscular strength" OR "hand/physiology" OR "strength, muscle" OR "muscle force" OR "muscle-strength" | 77,206 |
|  | #2 | "metabolic syndrome" OR "metabolic syndromes" OR "syndrome metabolic" OR "syndromes metabolic" OR "metabolic syndrome x" OR "insulin resistance syndrome x" OR "syndrome x metabolic" OR "syndrome x insulin resistance" OR "metabolic x syndrome" OR "syndrome metabolic x" OR "x syndrome metabolic" OR "dysmetabolic syndrome x" OR "reaven syndrome x" OR "syndrome x reaven" OR "metabolic cardiovascular syndrome" OR "cardiovascular syndrome metabolic" OR "syndrome metabolic cardiovascular" OR "cardiometabolic syndrome" OR "cardiometabolic syndromes" OR "syndrome cardiometabolic" OR "metabolic abnormality" OR "metabolic abnormalities" OR "metabolic disorder" OR "insulin-resistance" OR "syndrome metabolic cardiovascular" OR "metabolic cardiovascular syndrome" OR " cardiovascular syndrome, metabolic" OR " syndrome, metabolic cardiovascular" | 171,281 |
|  | #3 | #1 and #2 | 815 |
| WOS | #1 | TS = (hand strength OR handgrip OR handgrip strength OR grip strength OR holding power OR hand strengths OR strength hand OR strengths hand OR grip OR grips OR grasp OR grasps OR hand grip OR grip power OR grip-strength OR muscle strength OR muscle strength Dynamometer OR grip dynamometer OR muscle Weakness OR muscular strength OR hand/physiology OR strength, muscle OR muscle force OR muscle-strength) | 175,684 |
|  | #2 | TS = (metabolic syndrome OR metabolic syndromes OR syndrome metabolic OR syndromes metabolic OR metabolic syndrome x OR insulin resistance syndrome x OR syndrome x metabolic OR syndrome x insulin resistance OR metabolic x syndrome OR syndrome metabolic x OR x syndrome metabolic OR dysmetabolic syndrome x OR reaven syndrome x OR syndrome x reaven OR metabolic cardiovascular syndrome OR cardiovascular syndrome metabolic OR syndrome metabolic cardiovascular OR cardiometabolic syndrome OR cardiometabolic syndromes OR syndrome cardiometabolic OR metabolic abnormality OR metabolic abnormalities OR metabolic disorder OR insulin-resistance OR syndrome metabolic cardiovascular OR metabolic cardiovascular syndrome OR cardiovascular syndrome, metabolic OR syndrome, metabolic cardiovascular) | 327,919 |
|  | #3 | #1 and #2 | 2,247 |
| Embase | #1 | 'hand strength' OR 'handgrip' OR 'handgrip strength' OR 'holding power' OR 'hand strengths' OR 'strength hand' OR 'strengths hand' OR 'grip' OR 'grips' OR 'grasp' OR 'grasps' OR 'hand grip' OR 'grip power' OR 'grip strength' OR 'muscle strength' OR 'muscle strength dynamometer' OR 'grip dynamometer' OR 'muscle weakness' OR 'muscular strength' OR 'hand/physiology' OR 'strength,muscle' OR 'muscle force' OR 'muscle-strength' | 338,989 |
|  | #2 | 'metabolic syndrome' OR 'metabolic syndromes' OR 'syndrome metabolic' OR 'syndromes metabolic' OR 'metabolic syndrome x' OR 'insulin resistance syndrome x' OR 'syndrome x metabolic' OR 'syndrome x insulin resistance' OR 'metabolic x syndrome' OR 'syndrome metabolic x' OR 'x syndrome metabolic' OR 'dysmetabolic syndrome x' OR 'reaven syndrome x' OR 'syndrome x reaven' OR 'cardiovascular syndrome metabolic' OR 'cardiometabolic syndrome' OR 'cardiometabolic syndromes' OR 'syndrome cardiometabolic' OR 'metabolic abnormality' OR 'metabolic abnormalities' OR 'metabolic disorder' OR 'insulin-resistance' OR 'syndrome metabolic cardiovascular' OR 'metabolic cardiovascular syndrome' OR 'cardiovascular syndrome, metabolic' OR 'syndrome, metabolic cardiovascular' | 513,401 |
|  | #3 | #1 and #2 | 5,014 |
| CNKI | #1 | Handgrip strength | 58,352 |
|  | #2 | Metabolic syndrome | 133,103 |
|  | #3 | #1 and #2 | 1,153 |
| Wanfang | #1 | Handgrip strength | 8,427 |
|  | #2 | Metabolic syndrome | 48,574 |
|  | #3 | #1 and #2 | 36 |
| SinoMed | #1 | Handgrip strength | 3,700 |
|  | #2 | Metabolic syndrome | 15,991 |
|  | #3 | #1 and #2 | 7 |
