# Supplementary material for: Association between handgrip strength and metabolic syndrome: A meta-analysis and systematic review
Source: Front Nutr. 2022 Dec 1;9:996645. doi: 10.3389/fnut.2022.996645 (PMC9751936; doi:10.3389/fnut.2022.996645)
Supplement: Supplementary Figure 1 — Adjusted effect size using trim and fill method for HGS and MetS. [file Data_Sheet_1.ZIP › Supplementary material/Table S2. The quality assessment of the included cohort studies.docx]

**Table S2.** **The quality assessment of the included cohort studies**

| Study | Selection | | | | Comparability† | Outcome | | | Scores |
| --- | --- | --- | --- | --- | --- | --- | --- | --- | --- |
|  | representativeness of the exposed cohort | selection of the non-exposed cohort | ascertainment of exposure to implants | demonstration that outcome of interest was no present at start of study | control  important factors | assessment of outcome | follow-up long enough for outcomes to occur‡ | adequacy of follow up of cohort § |  |
| Shen, C. 2020(1) | * | * | * | * | ** | * | * | * | 9 |
| Jeon, Yoo Jeong 2021(2) | * | * | * | * | * | * | * | - | 7 |

*A study could be awarded a maximum of one star for each item except for the item Control for important factor or additional factor.

† A maximum of 2 stars could be awarded for this item. Studies that controlled for body mass index received one star, whereas studies that controlled for other important confounders such as age, Socio-demography et al received an additional star.

‡ A cohort study with a follow-up time >5 years was assigned one star.

§ A cohort study with a follow-up rate >85% was assigned one star.

**References**

1. Shen C, Lu J, Xu Z, Xu Y, Yang Y. Association between handgrip strength and the risk of new-onset metabolic syndrome: a population-based cohort study. BMJ Open. 2020;10(10):e041384.

2. Jeon YJ, Lee SK, Shin C. Relative Hand Grip and Back Muscle Strength, but Not Mean Muscle Strength, as Risk Factors for Incident Metabolic Syndrome and Its Metabolic Components: 16 Years of Follow-Up in a Population-Based Cohort Study. Applied Sciences-Basel. 2021;11(11).
