# Supplementary material for: Association between handgrip strength and metabolic syndrome: A meta-analysis and systematic review
Source: Front Nutr. 2022 Dec 1;9:996645. doi: 10.3389/fnut.2022.996645 (PMC9751936; doi:10.3389/fnut.2022.996645)
Supplement: Supplementary Figure 1 — Adjusted effect size using trim and fill method for HGS and MetS. [file Data_Sheet_1.ZIP › Supplementary material/Table S3. The quality assessment of the included cross-sectional studies.docx]

Criteria

Q1. Define the source of information (survey, record review)

Q2. List inclusion and exclusion criteria for exposed and unexposed subjects (cases and controls) or refer to previous publications

Q3. Indicate time period used for identifying patients

Q4. Indicate whether or not subjects were consecutive if not population-based

Q5. Indicate if evaluators of subjective components of study were masked to other aspects of the status of the participants

Q6. Describe any assessments undertaken for quality assurance purposes (e.g., test/retest of primary outcome measurements)

Q7. Explain any patient exclusions from analysis

Q8. Describe how confounding was assessed and/or controlled.

Q9. If applicable, explain how missing data were handled in the analysis

Q10. Summarize patient response rates and completeness of data collection

Q11. Clarify what follow-up, if any, was expected and the percentage of patients for which incomplete data or follow-up was obtained

**Table S3. The quality assessment of the included cross-sectional studies**

| Study | Q1 | Q2 | Q3 | Q4 | Q5 | Q6 | Q7 | Q8 | Q9 | Q10 | Q11 | Total |
| --- | --- | --- | --- | --- | --- | --- | --- | --- | --- | --- | --- | --- |
| Sayer, A. A. 2007(1) | 1 | 1 | 1 | 1 | 1 | 1 | 0 | 1 | 0 | 1 | 1 | 9 |
| Atlantis, E. 2009(2) | 1 | 0 | 1 | 1 | 1 | 1 | 1 | 0 | 0 | 1 | 0 | 7 |
| Ishii, Shinya 2014(3) | 1 | 0 | 1 | 1 | 1 | 0 | 0 | 1 | 1 | 1 | 0 | 7 |
| Chang, Ke-Vin 2015(4) | 1 | 1 | 1 | 1 | 1 | 0 | 0 | 1 | 0 | 0 | 0 | 6 |
| Kawamoto, R. 2016(5) | 1 | 0 | 0 | 1 | 1 | 0 | 0 | 1 | 0 | 0 | 0 | 4 |
| Byeon, J. Y. 2019(6) | 1 | 0 | 1 | 1 | 1 | 1 | 0 | 1 | 0 | 0 | 0 | 6 |
| Wu, H. 2019(7) | 1 | 1 | 1 | 1 | 1 | 1 | 0 | 1 | 1 | 0 | 0 | 8 |
| Chang, Ke-Vin 2019(8) | 1 | 1 | 1 | 1 | 1 | 0 | 0 | 1 | 0 | 0 | 0 | 6 |
| Mesinovic, J. 2019(9) | 1 | 0 | 1 | 1 | 1 | 1 | 0 | 1 | 0 | 0 | 0 | 6 |
| Merchant, R. A. 2020(10) | 1 | 1 | 1 | 1 | 1 | 0 | 0 | 1 | 1 | 1 | 0 | 8 |
| Ji, C. 2020(11) | 1 | 0 | 1 | 1 | 1 | 1 | 0 | 1 | 0 | 1 | 0 | 7 |
| Song, P. 2020(12) | 1 | 1 | 1 | 1 | 1 | 0 | 1 | 1 | 1 | 1 | 0 | 9 |
| Moreira, Mayle Andrade 2020(13) | 1 | 1 | 1 | 1 | 1 | 0 | 1 | 1 | 1 | 1 | 0 | 9 |
| Kim, H. 2020(14) | 1 | 1 | 1 | 1 | 1 | 0 | 0 | 1 | 0 | 0 | 0 | 6 |
| Wang, Y. 2020 | 1 | 1 | 1 | 1 | 1 | 1 | 0 | 1 | 0 | 0 | 0 | 7 |
| Kim, Ji-Su 2021(15) | 1 | 1 | 1 | 0 | 1 | 0 | 0 | 1 | 1 | 1 | 0 | 7 |
| Zhang, W. 2021(16) | 1 | 1 | 1 | 1 | 1 | 0 | 1 | 1 | 0 | 0 | 0 | 7 |

**References**

1. Sayer AA, Syddall HE, Dennison EM, Martin HJ, Phillips DIW, Cooper C, et al. Grip strength and the metabolic syndrome: findings from the Hertfordshire Cohort Study. Qjm-an International Journal of Medicine. 2007;100(11):707-13.

2. Atlantis E, Martin SA, Haren MT, Taylor AW, Wittert GA, Florey Adelaide Male Ageing S. Inverse associations between muscle mass, strength, and the metabolic syndrome. Metabolism-Clinical and Experimental. 2009;58(7):1013-22.

3. Ishii S, Tanaka T, Akishita M, Ouchi Y, Tuji T, Iijima K, et al. Metabolic Syndrome, Sarcopenia and Role of Sex and Age: Cross-Sectional Analysis of Kashiwa Cohort Study. Plos One. 2014;9(11).

4. Chang K-V, Hung C-Y, Li C-M, Lin Y-H, Wang T-G, Tsai K-S, et al. Reduced Flexibility Associated with Metabolic Syndrome in Community-Dwelling Elders. Plos One. 2015;10(1).

5. Kawamoto R, Ninomiya D, Kasai Y, Kusunoki T, Ohtsuka N, Kumagi T, et al. Handgrip strength is associated with metabolic syndrome among middle-aged and elderly community-dwelling persons. Clin Exp Hypertens. 2016;38(2):245-51.

6. Byeon JY, Lee MK, Yu M-S, Kang MJ, Lee DH, Kim KC, et al. Lower Relative Handgrip Strength is Significantly Associated with a Higher Prevalence of the Metabolic Syndrome in Adults. Metabolic Syndrome and Related Disorders. 2019;17(5):280-8.

7. Wu H, Liu M, Chi VTQ, Wang J, Zhang Q, Liu L, et al. Handgrip strength is inversely associated with metabolic syndrome and its separate components in middle aged and older adults: a large-scale population-based study. Metabolism. 2019;93:61-7.

8. Chang K-V, Yang K-C, Wu W-T, Huang K-C, Han D-S. Association between metabolic syndrome and limb muscle quantity and quality in older adults: a pilot ultrasound study. Diabetes Metabolic Syndrome and Obesity-Targets and Therapy. 2019;12:1821-30.

9. Mesinovic J, McMillan LB, Shore-Lorenti C, De Courten B, Ebeling PR, Scott D. Metabolic syndrome and its associations with components of sarcopenia in overweight and obese older adults. Journal of Clinical Medicine. 2019;8(2).

10. Merchant RA, Chan YH, Lim JY, Morley JE. Prevalence of Metabolic Syndrome and Association with Grip Strength in Older Adults: Findings from the HOPE Study. Diabetes Metab Syndr Obes. 2020;13:2677-86.

11. Ji C, Xia Y, Tong S, Wu Q, Zhao Y. Association of handgrip strength with the prevalence of metabolic syndrome in US adults: the national health and nutrition examination survey. Aging (Albany NY). 2020;12(9):7818-29.

12. Song P, Zhang Y, Wang Y, Han P, Fu L, Chen X, et al. Clinical relevance of different handgrip strength indexes and metabolic syndrome in Chinese community-dwelling elderly individuals. Arch Gerontol Geriatr. 2020;87:104010.

13. Moreira MA, Vafaei A, da Camara SMA, do Nascimento RA, de Morais MdSM, Almeida MdG, et al. Metabolic syndrome (MetS) and associated factors in middle-aged women: a cross-sectional study in Northeast Brazil. Women & Health. 2020;60(6):601-17.

14. Kim H, Kim YH, Kim W. Association of low muscle mass and isokinetic strength with metabolic syndrome. Journal of Men's Health. 2020;16(2):e50-e8.

15. Kim J-S, Seo Y. Relationship Between Handgrip Strength and Metabolic Syndrome Among Middle-Aged and Elderly Cancer Survivors: A National Population Study. Cancer nursing. 2021.

16. Zhang W, Zhao Z, Sun X, Tian X. Prevalence of metabolic syndrome according to absolute and relative values of muscle strength in middle-aged and elderly women. International Journal of Environmental Research and Public Health. 2021;18(17).
