# Supplementary material for: Association between handgrip strength and metabolic syndrome: A meta-analysis and systematic review
Source: Front Nutr. 2022 Dec 1;9:996645. doi: 10.3389/fnut.2022.996645 (PMC9751936; doi:10.3389/fnut.2022.996645)
Supplement: Supplementary Figure 1 — Adjusted effect size using trim and fill method for HGS and MetS. [file Data_Sheet_1.ZIP › Supplementary material/Table S4. Meta-regression analysis of HGS and MetS.docx]

**Table S4.** **Meta-regression analysis of HGS and MetS**

| **Variable** | **Coefficient** | **Standard error** | **P value** | **95% CI** |
| --- | --- | --- | --- | --- |
| Sex |  |  |  |  |
| Women (ref) |  |  |  |  |
| Men | 0.27 | 0.23 | 0.240 | -0.19, 0.74 |
| Diagnosed criteria of MetS |  |  |  |  |
| The joint scientific statement of harmonizing the MetS criteria (ref) |  |  |  |  |
| AHA | -0.79 | 0.43 | 0.079 | -1.68, 0.10 |
| ATP III | -1.36 | 0.37 | 0.001 | -2.13, -0.60 |
| IDF | -1.28 | 0.50 | 0.016 | -2.31, -0.26 |
| Chinese Diabetes Society | -0.88 | 0.48 | 0.081 | -1.88, 0.12 |
| HGS adjusted method |  |  |  |  |
| HGS adjusted by BMI (ref) |  |  |  |  |
| Absolute HGS | -0.44 | 0.43 | 0.314 | -1.31, 0.44 |
| HGS adjusted by weight | 0.22 | 0.36 | 0.552 | -0.53, 0.97 |
| Country Status |  |  |  |  |
| Developing country (ref) |  |  |  |  |
| Developed country | 0.51 | 0.22 | 0.025 | 0.07, 0.95 |
| Study design |  |  |  |  |
| Cross-sectional study (ref) |  |  |  |  |
| Cohort study | -0.32 | 0.32 | 0.325 | -0.97, 0.33 |
| Age |  |  |  |  |
| Elderly (ref) |  |  |  |  |
| Middle aged | -0.32 | 0.42 | 0.466 | -1.29, 0.65 |
| Measuring tools of HGS |  |  |  |  |
| Jamar (ref) |  |  |  |  |
| Takei | 0.85 | 0.35 | 0.024 | 0.13, 1.58 |
| CAMRY | 1.08 | 0.46 | 0.029 | 0.12, 2.04 |
| WCS-100 | 0.60 | 0.41 | 0.153 | -0.24, 1.44 |
| Publication year |  |  |  |  |
| Articles before 2016 (ref) |  |  |  |  |
| Articles after 2016 | 0.03 | 0.29 | 0.913 | -0.55, 0.62 |
| MetS for each component |  |  |  |  |
| Elevated triglycerides (ref) |  |  |  |  |
| Elevated waist circumference | 0.84 | 0.19 | 0.000 | 0.46, 1.23 |
| Low high-density lipoprotein-cholesterol | -0.08 | 0.19 | 0.685 | -0.47, 0.31 |
| Elevated blood pressure | -0.08 | 0.19 | 0.697 | -0.47, 0.32 |
| Elevated fasting blood glucose | -0.08 | 0.19 | 0.672 | -0.48, 0.31 |
