# Supplementary figures and images for: Association between handgrip strength and metabolic syndrome: A meta-analysis and systematic review
Source: Front Nutr. 2022 Dec 1;9:996645. doi: 10.3389/fnut.2022.996645 (PMC9751936; doi:10.3389/fnut.2022.996645)

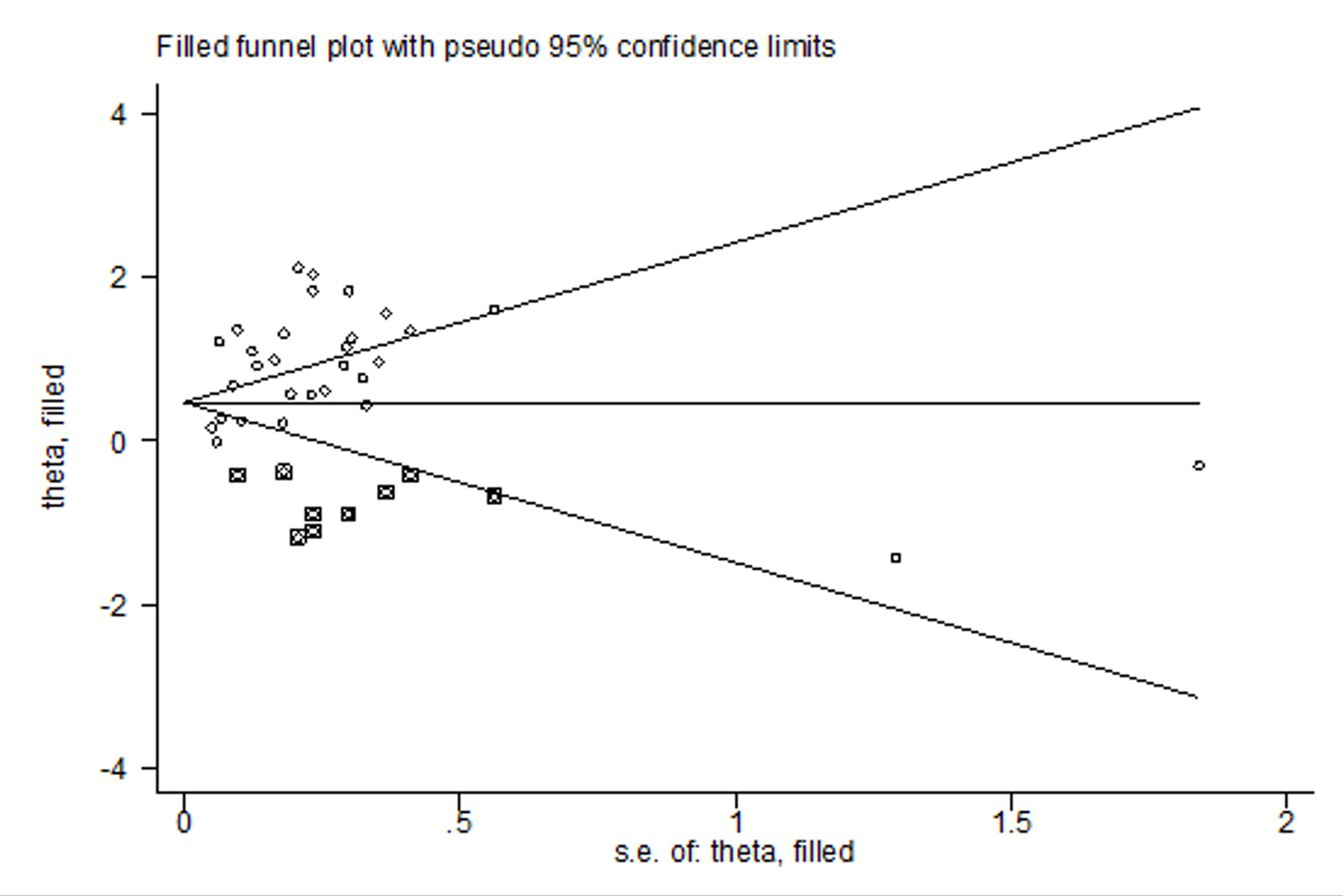

Supplement: Supplementary Figure 1 — Adjusted effect size using trim and fill method for HGS and MetS. [file Data_Sheet_1.ZIP › Supplementary material/Figure S1. Adjusted effect size using trim and fill method for HGS and MetS.jpg]
